# Oleuropein-Rich Jasminum Grandiflorum Flower Extract Regulates the LKB1-PGC-1α Axis Related to the Attenuation of Hepatocellular Lipid Dysmetabolism

**DOI:** 10.3390/nu16010058

**Published:** 2023-12-24

**Authors:** Yajun Hou, Xuan Zhao, Yalin Wang, Yapeng Li, Caihong Chen, Xiu Zhou, Jingwei Jin, Jiming Ye, Dongli Li, Lishe Gan, Rihui Wu

**Affiliations:** 1 School of Pharmacy and Food Engineering, Wuyi University, Jiangmen 529020, China; hyjhyj7083@163.com (Y.H.); 19520415561@163.com (X.Z.); wangyalin13@foxmail.com (Y.W.); liyapengsw@126.com (Y.L.); 18138087910@163.com (C.C.); zhou.xiu@hotmail.com (X.Z.); wyuchemjjw@126.com (J.J.); jiming_ye1@163.com (J.Y.); wyuchemldl@126.com (D.L.); ganlishe@163.com (L.G.); 2 International Healthcare Innovation Institute (Jiangmen), Jiangmen 529040, China

**Keywords:** hepatic steatosis, oleuropein, *Jasminum grandiflorum*, LKB1-PGC-1α axis

## Abstract

Diets rich in fat are a major cause of metabolic disease, and nutritional food has been widely used to counteract the metabolic disorders such as obesity and fatty liver. The present study investigated the effects of oleuropein-enriched extract from *Jasminum grandiflorum* L. flowers (OLE-JGF) in high-fat diet (HFD)-fed mice and oleic acid (OA)-treated AML-12 cells. Treatment of HFD-fed mice with 0.6% OLE-JGF for 8 weeks significantly reduced body and liver weights, as well as attenuating lipid dysmetabolism and hepatic steatosis. OLE-JGF administration prominently suppressed the mRNA expressions of monocyte chemoattractant protein-1 (MCP-1) and cluster of differentiation 68 (CD68), and it also downregulated acetyl-CoA carboxylase (ACC) and fatty acid synthase (FAS) as well as sterol-regulatory-element-binding protein (SREBP-1c) in the liver. Meanwhile, mitochondrial DNA and uncoupling protein 2 (UCP2) were upregulated along with the increased expression of mitochondrial biogenic promoters including liver kinase B1 (LKB1), peroxisome proliferator–activated receptor-γ coactivator-1α (PGC-1α), nuclear factor-erythroid-derived 2-like 2 (Nrf2), and mitochondrial transcription factor A (Tfam), but did not change AMP-activated protein kinase (AMPK) in liver. The lipid droplets were decreased significantly after treatment with 80 μM oleuropein for 24 h in OA-induced AML-12 cells. Furthermore, oleuropein significantly inhibited ACC mRNA expression and upregulated LKB1, PGC-1α, and Tfam mRNA levels, as well as increasing the binding level of LKB1 to PGC-1α promoter in OA-induced cells. These findings indicate that OLE-JGF reduces hepatic lipid deposition in HFD-fed mice, as well as the fact that OA-induced liver cells may be partly attributed to upregulation of the LKB1-PGC-1α axis, which mediates hepatic lipogenesis and mitochondrial biogenesis. Our study provides a scientific basis for the benefits and potential use of the *J. grandiflorum* flower as a food supplement for the prevention and treatment of metabolic disease.

## 1. Introduction

Metabolic syndrome (MetS) is a constellation of multiple clinical health abnormalities, such as obesity, insulin resistance, hyperlipidemia, and hepatic steatosis. These pathological abnormalities may result in an increase in the risk of various cardiovascular diseases and certain types of cancer [1]. At least 30% of adults were found to be suffering from MetS in the Western world. A growing body of research has highlighted that lipid accumulation results from energy uptake exceeding energy expenditure, and the excess energy is mainly stored through the fatty acid synthesis and oxidation pathways [2,3]. However, the current effective interventions, especially therapeutic drugs approved for MetS, are still not sufficient, and their therapeutic effect remains unsatisfactory in the long term [4]. Therefore, there is an urgent need for the development of effective and safe therapies including dietary supplements to reduce the risks associated with MetS. 

*Jasminum grandiflorum* L. is a plant of the *Oleaceae* family whose flowers and leaves have been widely used in traditional medicine for the treatment of liver injury, inflammatory bowel disease, and cancers [5,6,7]. Notably, our previous studies have identified ethanol extracts of the flowers of *J. grandiflorum* exert protective effects against tetradecanoylphorbol-acetate-induced ear edema, HCl/EtOH-induced gastric ulcer, and CCl_4_-induced liver injury mainly by reducing inflammation and oxidative stress in mice [5,8,9]. These strong activities may be attributed to the presence of bioactive compounds in the flower of *J. grandiflorum* L., including secoiridoids, terpenoids, flavonoids, and tannins [7]. Generally, these natural products and their derivatives represent a wide range of biological and pharmacological activities [10,11]. For example, oleuropein (OLE), one of the secoiridoids, has been suggested to attenuate high-fat diet (HFD)-induced hepatic steatosis in mice by regulating the Wnt10b- and FGFR1-mediated signaling cascades, along with the TLR2- and TLR4-mediated signaling [12]. Furthermore, OLE not only reduces free-fatty-acid-induced lipogenesis via lowered extracellular-signal-regulated kinase activation in hepatocytes [13] but also prevents the progression of HFD-induced steatohepatitis to hepatic fibrosis in mice [14]. Notably, some plants rich in OLE (such as olive) have been suggested to be beneficial for the prevention of MetS arising from obesity [15,16]. Therefore, we hypothesized that OLE-rich extract from flowers of *J. grandiflorum* (OLE-JGF) may offer a potentially beneficial effect on the regression of MetS, whereas the detailed molecular mechanism is less well understood.

In the present study, we investigated the effect of OLE-JGF on the prevention of MetS and its underlying mechanisms in HFD-fed mice and oleic acid (OA)-induced liver cells. Administration of OLE-JGF could effectively ameliorate obesity and related metabolic disorders, which may be associated with the inhibition of hepatic lipogenesis and stimulation of mitochondrial biogenesis controlled by the liver kinase B1 (LKB1)-PGC-1α axis.

## 2. Materials and Methods

### 2.1. Preparation of OLE-JGF

The extraction and purification of OLE-JGF were prepared by the standard process of organic solvent extraction and macroporous resin column chromatography [5,9]. In brief, air-dried and powdered *J. grandiflorum* flowers were extracted with aqueous ethanol (80%) at room temperature three times. The ethanol extract was combined and dried with a rotary evaporator under reduced pressure. The yield residue was then suspended in water and sequentially submitted to liquid–liquid partitioning with petroleum ether, ethyl acetate, and n-BuOH. A partial of the n-BuOH fraction was chromatographed over macroporous resin and eluted successively with a gradient aqueous ethanol system (0, 10, 20, and 30%). The 30% ethanol eluant was collected and combined to afford OLE-JGF based on thin-layer chromatography analysis. The OLE-JGF was qualitatively and quantitatively analyzed by high-performance liquid chromatography with diode-array detection (HPLC, Waters, MA, USA) at the wavelength of 280 nm.

### 2.2. Animals and Experimental Design

Six-week-old male C57BL/6J mice were purchased from the Guangdong Medical Laboratory Animal Center (Foshan, China). Mice were maintained under standard management conditions (temperature of 25 ± 2 °C and a 12 h light–dark cycle) and performed in accordance with the procedures approved by the Animal Care and Ethics Committee of Wuyi University (Jiangmen, China; Approval code: CN2021010). After 2 weeks of acclimatization, mice were randomly assigned into three groups. They were fed with chow diet (11% calories from fat, 23% calories from protein, and 65% calories from carbohydrate) or HFD (45% calories from fat, 20% calories from protein, and 35% calories from carbohydrate) with or without 0.6% OLE-JGF (HFD-JGF) ad libitum for 8 weeks. Diets were stored at −20° C and changed twice a week during the experiments. Food intake and body weight were monitored weekly.

### 2.3. Cell and Treatment

Alpha mouse liver 12 (AML-12) cells were originally purchased from American Type Culture Collection (ATCC, Manassas, VA, USA). Cells were cultured in high-glucose Dulbecco’s modified Eagle’s medium (DMEM, Gibco, New York, NY, USA) and Ham’s F-12 (1:1; Gibco, New York, NY, USA) supplemented with 10% fetal bovine serum (FBS), 100 U/mL penicillin and 100 μg/mL streptomycin (Gibco, New York, NY, USA), 1% insulin transferrin selenium solution (Sigma, St. Louis, MO, USA), and 40 ng/mL dexamethasone (Sigma, St. Louis, MO, USA). After 24 h incubation, cells were treated with 0.25 mM OA (dissolved in 5% BSA) for another 24 h in high-glucose, serum-free DMEM and Ham’s F-12 (1:1), followed by the administration of OLE (80 μM) or metformin (10 μM, LKB1 activator) (Sigma, St. Louis, MO, USA) for 24 h.

### 2.4. Biochemical Analysis

The biochemical indicators were detected as described by previous description with slight modification [17,18]. Serum samples were collected from 12 h fasted mice at the end of the experiment. Serum total triglyceride (TG), cholesterol (TC), low-density lipoprotein cholesterol (LDL-C), alanine aminotransferase (ALT), and aspartate transaminase (AST) were measured using commercially available kits (Biosino Bio-Technology and Science Inc., Beijing, China) following the manufacturer’s instructions on a FAITH-1000 automatic biochemistry analyzer (Nanjing Laura Electronics Co., Ltd., Nanjing, China).

### 2.5. Determination of TG in Liver Tissue and Cells

Liver TG was extracted and determined as previously described [18]. Briefly, 20–30 mg liver tissues were lysed with 400 μL 0.5 M KOH, homogenized and incubated at 60 °C for 10 min, then centrifuged at 3000 rpm for 15 min. A total of 5 μL supernatant per sample was added into 300 μL TG GPO-PAP reagent (Nanjing Jiancheng Bioengineering Institute, Nanjing, China) and incubated at 37 °C for 10 min. Optical density (OD) values at 510 nm were measured using a microplate reader (Synergy neo2; Biotek Instruments, Winooski, VT, USA). The TG contents were calculated according to the standard curve equation: y = 3.2727x − 0.2239, R^2^ = 0.9958 (Figure S1A).

For the determination of cellular TG level, cells were collected and homogenized using an ultrasonic cell crusher (Thermo Fisher Scientific, Waltham, MA, USA) at 300 W for five times (5 s each time). A total of 20 μL cell homogenate was added into 300 μL TG GPO-PAP reagent (Nanjing Jiancheng Bioengineering Institute, Nanjing, China). The suspension was then incubated at 37 °C for 10 min, and the OD value at 510 nm was measured using a microplate reader (Syn-ergy neo2; Biotek Instruments, Winooski, VT, USA). The TG contents were calculated according to the standard curve equation: y = 2.6161x − 0.1319, R^2^ = 0.9990 (Figure S1B).

### 2.6. Histological Analysis

Hematoxylin and eosin (H&E) staining was performed according to standard method. For the paraffin section, liver was fixed in 4% paraformaldehyde (Biosharp, Hefei, China). After 24 h fixation, the tissue was dehydrated and embedded in paraffin. The tissue was then cut into 5 μm slices using a Leica RM22559 microtome (Leica, Wetzlar, Germany). The section was dewaxed, hydrated, and then stained with hematoxylin and eosin. The images were captured using an Olympus BX41 microscope (Olympus Co., Ltd., Tokyo, Japan).

For oil red O staining, cells were fixed with 4% paraformaldehyde at room temperature for 1 h and washed three times with phosphate-buffered solution (PBS, Gibco, New York, NY, USA). Cells were then stained with 0.3% oil red O (Sigma, St. Louis, MO, USA) solution for 1 h at room temperature. After rinsing with PBS, the cells were snapped with an Olympus BX41 microscope (Olympus Co., Ltd., Tokyo, Japan).

### 2.7. Western Blot

The expressions of proteins were measured using Western blot as described before [19,20]. In brief, total protein of liver or cells was extracted with RIPA lysis buffer and quantified using a BCA protein assay (Thermo Fisher Scientific, Waltham, MA, USA). Protein sample was prepared in Laemmli buffer, and 20 µL of sample was added and separated by 10% SDS-PAGE gel. The proteins were transferred onto Immobilon^®^-P transfer membranes at 90 V for 90 min. After blocking with 3% BSA in TBST for 1 h at room temperature, membranes were incubated with the following primary antibodies: glyceraldehyde 3-phosphate dehydrogenase (1:1000; GAPDH, Cell Signaling, Danvers, MA, USA), total-AMPK, and phospho-AMPKα1 (Ser 496) rabbit monoclonal antibody (1:1000; Beyotime, Shanghai, China) at 4 °C for 12 h. Then, membranes were incubated with secondary antibodies (1:3000; Beyotime, Shanghai, China) for 2 h at a room temperature. The protein bands of interest were detected by BeyoECL Plus (Beyotime, Shanghai, China). Images of the membranes were taken with the GeIView 1500 system (Guangzhou, China). The intensities of protein bands were analyzed using Image Lab software (version 6.1) and the values were normalized to GAPDH.

### 2.8. Quantitative RT-PCR

Hepatic and cellular gene expressions were analyzed as described in our previous report [21]. Briefly, total RNA of the liver or cell was extracted by using RNAiso Plus (Takara Bio., Ltd., Kusatsu, Japan) and dissolved in RNase-free water. The concentration of RNA was measured by NanoDrop One/One (Thermo Fisher Scientific, Waltham, MA, USA). The RNA was thermally denatured for 5 min at 65 °C and immediately placed on ice to cool down. After adding the ReverTra Ace^TM^ qPCR RT Master Mix (Toyobo Co., Ltd., Osaka, Japan), the sample was placed in a PCR apparatus for reverse transcription at 37 °C for 15 min and 50 °C for 5 min. The enzyme inactivation reaction was performed at 98 °C for 5 min. Then, the sample was cooled at 4 °C. SYBR Green qPCR SuperMix (Toyobo Co., Ltd., Osaka, Japan) was used to amplify the targeted genes using three-step annealing on the LightCycler^®^ 96 real-time PCR system according to the manufacturer’s instructions. The targeted genes include fatty acid synthase (FAS), sterol regulatory element-binding protein (SREBP-1c), acetyl-CoA carboxylase (ACC), LKB1, peroxisome proliferator–activated receptor-γ coactivator-1α (PGC-1α), nuclear-factor-erythroid-derived 2-like 2 (Nrf2), mitochondrial transcription factor A (Tfam), mitochondrial DNA (mtDNA), uncoupling protein 2 (UCP2), cluster of differentiation 68 (CD68), monocyte chemoattractant protein-1 (MCP-1), tumor necrosis factor-α (TNF-α), and β-actin. Relative expression levels were normalized against the endogenous house-keeping gene β-actin. Sequences of the real-time PCR primers used are listed in Table S1.

### 2.9. Determination of Mitochondrial DNA (mtDNA) Copy Numbers

Quantification of mtDNA copy numbers was performed by PCR as previously described [22]. Briefly, the total DNA of the liver sample was extracted using a DNA extraction kit (Beyotime, Shanghai, China). A total of 25 mg of liver tissue was homogenized using lysate A with protease K and incubated at 55 °C water for 1 h. The sample was kept at room temperature for 2 min after adding RNase A to remove RNA. Lysate B was added before incubation at 70 °C for 10 min, and then anhydrous ethanol was added. The mixture was transferred to DNA purification column, washed and centrifuged with washing solution, and eluted with eluent. The concentration of DNA was measured by NanoDrop One/One (Thermo Fisher Scientific, Waltham, MA, USA). The qPCR was performed to determine the copy numbers of nuclear DNA (nDNA) and mtDNA using primers targeted toward the cytochrome B gene (for mtDNA) and 18S rRNA (for nDNA). The primers used are listed in Table S2.

### 2.10. Chromatin Immunoprecipitation (ChIP)

ChIP detection was performed using a Pierce Agarose ChIP Kit (Thermo Fisher Scientific, Waltham, MA, USA) according to the manufacturer’s instructions. In brief, the minced tissue or cells were crosslinked with 1.5% formaldehyde prior to 0.125 M glycine treatment. The sample was then resuspended in cold PBS and washed three times and ground in PBS. The supernatants were diluted with dilution buffer (0.01% SDS, 1.1% Trition, 1.2 mM EDTA, 16.7 mM Tris-Cl (pH 8.0), 167 mM NaCl, 4.1 mM PMSF), and aliquots from each sample were saved as the input DNA for quantification of total amount DNA. Cross-linked LKB1 protein–DNA complexes were immunoprecipitated using LKB1-specific antibody (1:2000; Affinity, Cincinnati, OH, USA) or normal rabbit immunoglobulin G on a rocking platform at 4 °C overnight. ChIP-Grade Protein A/G Plus Agarose (Thermo Fisher Scientific, Waltham, MA, USA) was added, and the mixture was incubated for 1 h at 4 °C. Finally, IP elution and DNA recovery were performed, and the immunoprecipitated DNA was amplified by quantitative PCR with the PGC-1α promoter primer listed in Table S3.

### 2.11. Statistical Analysis

Data were presented as means ± SEM. Statistical analysis and visualization were performed using Prism 7 (GraphPad Software, La Jolla, CA, USA). Differences between groups were statistically analyzed using one-way ANOVA followed by Tukey’s multiple comparison tests unless indicated otherwise. *p* < 0.05 was considered statistically significant.

## 3. Results

### 3.1. OLE-JGF Preparation

**Figure 1:**
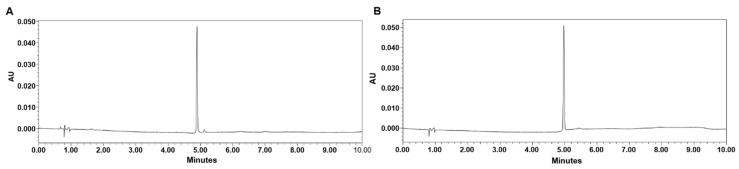
HPLC chromatograms of (**A**) OLE-JGF and (**B**) OLE standard solution. The X-axis represents retention time, and AU on the Y-axis represents absorbance units at the wavelength of 280 nm.

OLE is the main constituent in flowers of *J. grandiflorum* (JGF). After the extraction and purification, the total content of OLE in OLE-JGF was increased to 69% determined by HPLC. The HPLC chromatograms of OLE-JGF and OLE standard solution are shown in Figure 1A,B, respectively.

### 3.2. OLE-JGF Attenuated Liver Inflammation and Steatosis in HFD-Fed Mice

**Figure 2:**
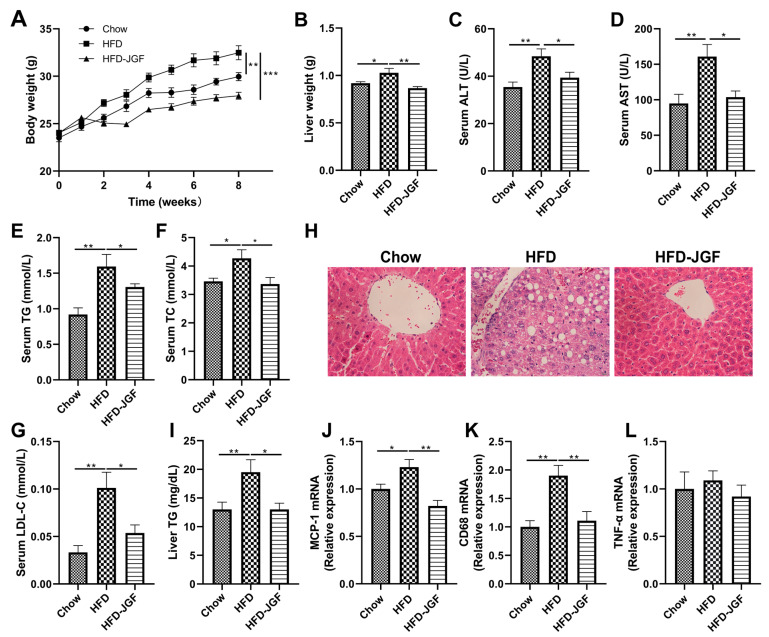
OLE-JGF reduced the fat deposition and hepatic steatosis in HFD-fed mice. Mice were randomly divided into three groups and treated with chow, HFD, or HFD-supplemented with 0.6% OLE-JGF for 8 weeks. (**A**) Body weight. (**B**) Liver weight. (**C**,**D**) The levels of ALT and AST in serum. (**E**–**G**) The serum levels of TG, TC, and LDL-C. (**H**) Representative images of H&E staining for the liver. (**I**) The contents of hepatic TG. (**J**–**L**) Relative mRNA expression of markers for MCP-1 and inflammatory cytokines (CD68 and TNF-α) in the liver. Data are presented as mean ± SEM (*n* = 6–8). Differences were assessed using one-way ANOVA test followed by multiple comparisons. * *p* < 0.05, ** *p* < 0.01, and *** *p* < 0.01.

To address whether OLE-JGF displays a metabolic protective effect on liver steatosis, mice were fed by HFD for 8 weeks with or without 0.6% OLE-JGF. Compared with the chow mice, HFD feeding induced a 12% increase in body weight (Figure 2A), which was significantly decreased after OLE-JGF treatment. HFD feeding caused a clear increase in the liver weight in mice, while supplementation with OLE-JGF significantly decreased liver weight in HFD-fed mice (Figure 2B). Higher levels of serum ALT and AST were found in HFD-fed mice than those of chow-fed mice and HFD-fed mice treated by OLE-JGF (Figure 2C,D), suggesting that administration of OLE-JGF exhibited protective effects against the HFD-induced liver damage in mice. Furthermore, OLE-JGF treatment significantly reduced serum TG, TC, and LDL-C levels and the hepatic TG level in HFD-fed mice when compared to the mice fed by HFD alone (Figure 2E–G,I). Consistent with these changes, HFD feeding resulted in a manifest increase in hepatocyte swelling and ballooning in the liver. These pathological changes were evidently mitigated by OLE-JGF (Figure 2H). We also observed that OLE-JGF treatment could potently reduce the mRNA expressions of MCP-1 and inflammatory cytokine CD68 in hepatic tissues, although these differences were not statistically significant in TNF-α mRNA expression (Figure 2J,K). These results indicate that OLE-JGF exhibits strong efficacy against fat deposition and hepatic steatosis induced by HFD in mice.

### 3.3. OLE-JGF Inhibited Hepatic Lipogenesis and Activated Mitochondrial Biogenesis in HFD-Fed Mice

**Figure 3:**
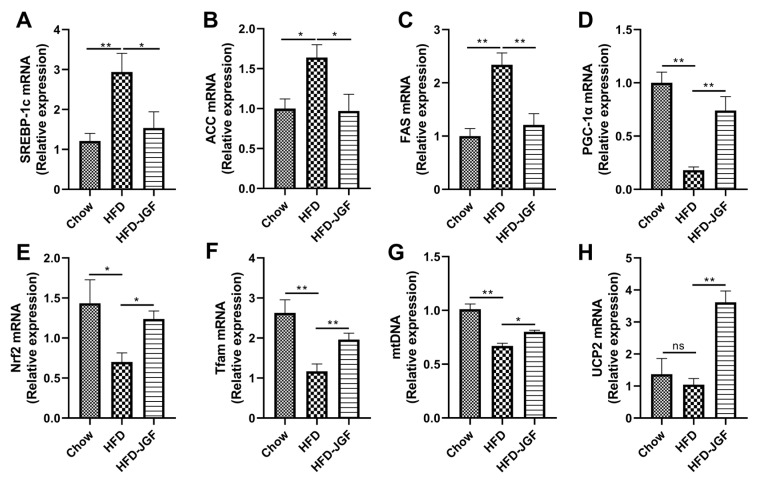
Effects of OLE-JGF on hepatic lipogenesis, mitochondrial biogenesis, and thermogenesis in HFD-fed mice. (**A**–**C**) Relative mRNA expression of hepatic-lipogenesis-related genes (**A**) SREBP-1c, (**B**) ACC, and (**C**) FAS in liver tissue. (**D**–**F**) Relative mRNA expression of mitochondrial-biogenesis-related genes: (**D**) PGC-1α, (**E**) Nrf2, and (**F**) Tfam. (**G**,**H**) Relative mRNA expression of markers for (**G**) the number of mitochondria (mtDNA) and (**H**) the thermogenesis-related gene (UCP2) in liver tissue. Data are presented as mean ± SEM (*n* = 6–8). Differences were assessed using one-way ANOVA test followed by multiple group comparisons. * *p* < 0.05 and ** *p* < 0.01; ns, not significance.

To investigate whether the anti-steatosis effect of OLE-JGF is related to the metabolic pathway of de novo lipogenesis, we next measured the regulators of fatty acid synthesis in the liver. HFD feeding for 8 weeks induced a significant increase in the mRNA expressions of SREBP-1c (≈2-fold) (Figure 3A), which resulted in an upregulation of its target lipogenic enzymes, including ACC (70%) (Figure 3B) and FAS (≈1-fold) (Figure 3C) compared with chow-fed mice. OLE-JGF supplementation significantly downregulated the mRNA expressions of SREBP-1c, ACC, and FAS expression in liver tissue (≈1-fold). We also determined mitochondrial-biogenesis-related genes, such as PGC-1α (Figure 3D), Nrf2 (Figure 3E), and Tfam (Figure 3F), to investigate whether OLE-JGF administration alleviates hepatic steatosis by regulating mitochondrial biogenesis and enhancing energy expenditure in mice. HFD feeding for 8 weeks decreased the hepatic mRNA expressions of PGC-1α (80%), Tfam (60%), and Nrf2 (70%) significantly compared with chow feeding. In agreement with these changes, the mRNA expression of mtDNA (Figure 3G) in HFD-fed mice was significantly decreased, but not the thermogenic gene mitochondrial UCP2 (Figure 3H). OLE-JGF treatment dramatically increased the mRNA expressions of hepatic PGC-1α (≈3-fold), Tfam (50%), Nrf2 (70%), mtDNA (20%), and UCP2 (≈2-fold) of HFD-fed mice. These data demonstrate that the anti-steatosis effect of OLE-JGF may be in part due to the regulation of the metabolic pathway of de novo lipogenesis and mitochondrial biogenesis and thermogenesis in HFD-induced obese mice. The decreased lipogenesis and increased energy expenditure contribute to the reduction of lipid accumulation.

### 3.4. OLE-JGF Activated Hepatic LKB1/PGC-1α Signaling Pathway in HFD-Fed Mice

**Figure 4:**
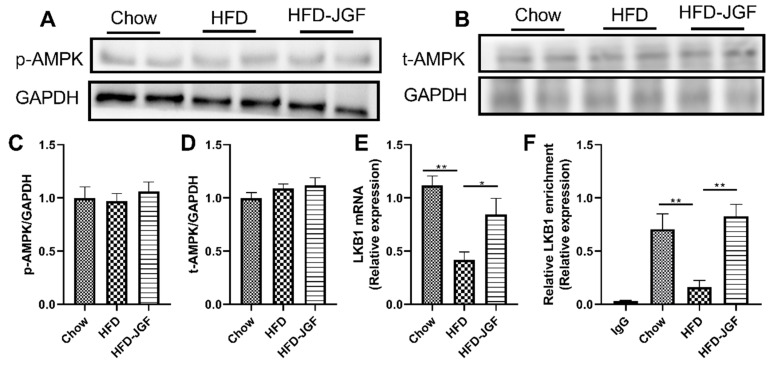
OLE-JGF upregulated LKB1 independent of AMPK in mice. (**A**–**D**) Representative images of p-AMPK/t-AMPK and quantified ratio of p-AMPK/t-AMPK to GAPDH. (**E**) The mRNA expression of hepatic LKB1. (**F**) LKB1 recruitment to the PGC-1α promoter region in OLE-JGF-treated mice detected by ChIP assay. IgG was used as a negative control. Data are presented as mean ± SEM (*n* = 4–6). * *p* < 0.05 and ** *p* < 0.01.

AMPK signaling pathways are potential targets for the treatment of metabolic disorders, including obesity, insulin resistance, and nonalcoholic fatty liver disease (NAFLD) [23,24]. Phosphorylation of AMPK regulates the cellular AMP/ATP ratio and activates the enzyme ACC, which provides substrates for fatty acid synthesis [24]. Therefore, we investigated whether OLE-JGF activate AMPK phosphorylation. OLE-JGF supplementation did not significantly alter the expressions of the total and phosphorylation of AMPK in HFD-treated mice (Figure 4A–D). We next tested the expression of LKB1, which is an upstream activator of AMPK. HFD administration significantly decreased LKB1 mRNA expression (60%) in liver tissue compared with chow-fed mice. OLE-JGF supplementation significantly upregulated LKB1 mRNA expression in liver tissue in mice fed an HFD (≈1-fold) (Figure 4E). To determine whether the activation of PGC-1α was LKB1 dependent, a ChIP test was performed for LKB1 and PGC-1α. It was found that HFD dramatically inhibited the binding of LKB1 to PGC-1α promoter (80%) in liver tissue compared with chow-fed mice. However, the LKB1 occupancy at the promoter of PGC-1α in liver was significantly enhanced by OLE-JGF supplementation (≈4-fold), suggesting OLE-JGF administration could activate LKB1 and a direct downstream target gene PGC-1α (Figure 4F). These findings indicate that OLE-JGF administration alleviates metabolic dysfunction with the involvement of LKB1/PGC-1α signaling pathway in an AMPK-independent manner.

### 3.5. OLE-JGF Inhibited OA-Induced Lipid Production in Liver Cells

**Figure 5:**
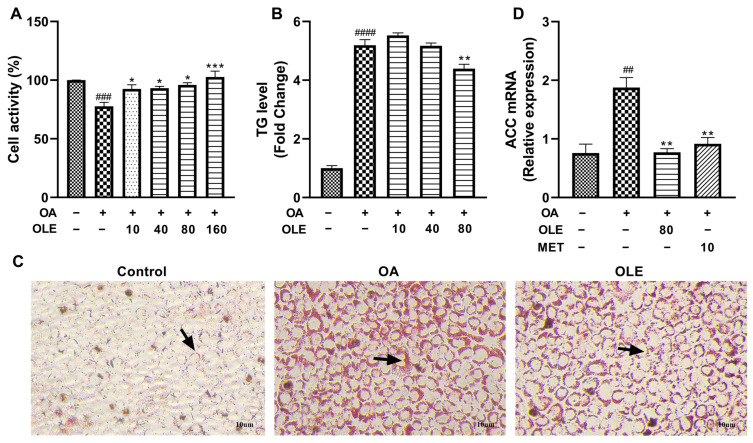
Effects of OLE on lipid production in OA-induced cells. (**A**) The activity of AML-12 cells treated with or without OA (0.25 µM) or different concentrations of OLE. (**B**) TG levels in AML-12 cells untreated or treated with OA or/and OLE. (**C**) Representative images of AML-12 cells stained with oil red O staining. (**D**) Relative mRNA expression of hepatic-lipogenesis-related genes (ACC) in cells. Data are presented as mean ± SEM (*n* = 3). ## *p* < 0.01, ### *p* < 0.001, #### *p* < 0.0001 showed compared to control; * *p* < 0.05, ** *p* < 0.01, *** *p* < 0.001 showed compared to cells treated with OA alone. Arrows indicated the formation of lipid droplets.

To confirm liver as the major site of OLE-JGF’s lipid-lowering effect on metabolism, an additional experiment in hepatocytes was conducted. The activity of AML-12 cells was significantly decreased after OA treatment when compared to controls. Compared with OA-induced cells, treatment with OLE dramatically increased the activity of cells treated by OA in a dose-dependent manner (Figure 5A). As shown in the Figure 5C of oil red O staining, the formation of lipid droplets in OA-induced cells was significantly increased compared to control cells, while lipid droplets were obviously reduced after OLE treatment. The results of cellular TG accumulation test showed a significant increase in the cells treated by 0.25 mM OA for 24 h, which was significantly inhibited by the treatment of 80 μM OLE (Figure 5B). Furthermore, OA induction significantly upregulated the mRNA expression of ACC (≈1-fold), which was effectively downregulated after supplementation with both OLE and LKB1 activator-metformin (≈1-fold) (Figure 5D). These results indicate that OLE exhibits strong efficacy against lipid production with the activation of LKB1 in OA-induced cells.

### 3.6. OLE Stimulated PGC-1α Involved in Mitochondrial Biogenesis in OA-Induced Liver Cells

**Figure 6:**
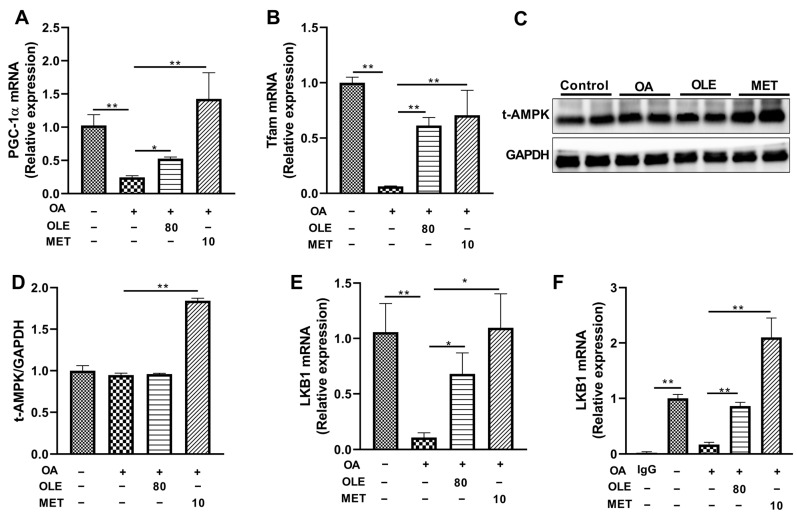
OLE-enhanced mitochondrial biogenesis was related to the LKB1-PGC-1α pathway in OA-induced cells. Relative mRNA expression of mitochondrial-biogenesis-related genes (**A**) PGC-1α and (**B**) Tfam. (**C**,**D**) Representative band of t-AMPK and relative expression of t-AMPK to GAPDH. (**E**) Relative mRNA expression of LKB1. (**F**) LKB1 recruitment to the PGC-1α promoter region in OLE-JGF-treated mice liver cells detected by ChIP assay. IgG was used as a negative control. Two-tailed unpaired *t*-test was used to analyze the mRNA expression of PGC-1α and LKB1. Data are presented as mean ± SEM (*n* = 4–6). * *p* < 0.05 and ** *p* < 0.01.

To further confirm the underlying mechanism of the lipid-lowering effect of OLE-JGF, mitochondrial-biogenesis-related genes including PGC-1α and Tfam were determined in OA-induced cells. Compared with control cells, OA exposed to cells for 24 h significantly decreased the mRNA expressions of PGC-1α (80%) and Tfam (90%) (Figure 6A,B). Accordingly, the mRNA expressions of PGC-1α (≈1-fold) and Tfam (≈6-fold) were dramatically upregulated after treatment with OLE in OA-induced cells. Also, the mRNA expressions of PGC-1α and Tfam were significantly increased by metformin (a LKB1 activator) supplementation with activating LKB1 in OA-treated cells (Figure 6A,B). Furthermore, compared with OA-treated cells, OLE supplementation was unable to alter the expressions of the t-AMPK in OA-treated cells, but the expressions of t-AMPK could be significantly upregulated by metformin treatment (Figure 6C,D). These data suggest that the lipid-lowering effect of OLE may be in part due to the regulation of mitochondrial biogenesis and thermogenesis in OA-induced cells, which was independent of AMPK signaling.

### 3.7. The Activation of LKB1-PGC-1α Pathway Participated in the Lipid-Lowering Effect of OLE in OA-Induced Liver Cells

To test how the LKB1 partakes in the inhibition of lipid production of OLE in cells, the mRNA expressions of LKB1 and PGC-1α were examined in OA-induced liver cells. As shown in Figure 6E, administration of OA significantly decreased LKB1 mRNA expression (80%) in cells compared with that of controls, suggesting that OA may be an effective LKB1 inhibitor. Interestingly, LKB1 mRNA expression was significantly upregulated by treatment with both OLE (≈2-fold) and metformin (≈4-fold) in OA-induced liver cells (Figure 6E). To determine whether the activation of PGC-1α was LKB1 dependent, a ChIP test was performed for LKB1 and PGC-1α. It was found that OA dramatically inhibited the binding of LKB1 to the PGC-1α promoter (80%) in cells compared with control cells (Figure 6F). However, the LKB1 occupancy at the promoter of PGC-1α in liver cells was significantly enhanced by OLE (≈3-fold) and LKB1 activator metformin (≈10-fold) (Figure 6F). These results suggest that the activation LKB1 and its direct downstream target gene PGC-1α participates in the process of lipid-lowering effect of OLE administration.

## 4. Discussion

Alterations in hepatic lipid metabolism have been suggested to contribute to the development of the most frequent metabolic disorders, such as NAFLD, obesity, and type 2 diabetes mellitus [3]. There is still lack effective therapies, because the progression of lipid dysmetabolism is tightly associated with inflammation and complex metabolic reprogramming. Several previous studies have indicated that supplementation of herbal remedies or natural products could effectively alleviate the severity of metabolic disease [25,26]. The present study provides evidence that consumption of oleuropein (OLE)-rich extract from the flowers of *J. grandiflorum* (OLE-JGF) effectively protected against the development of HFD-induced liver steatosis, low-grade inflammation, and related metabolic dysfunctions in mice. Furthermore, OLE-JGF supplementation was able to excellently restrain hepatic lipogenesis, accelerate fatty acid oxidation, and stimulate mitochondrial biogenesis and thermogenesis in the liver, which might be responsible for the function of OLE-JGF on the improvement of lipid and glucose metabolism, as well as energy consumption, in HFD-fed mice. Furthermore, treatment of OLE effectively regulates the activation of the LKB1-PGC-1α pathway involved the inhibition of lipid production and mitochondrial biogenesis in OA-induced liver cells. The OLE-JGF might be a promising therapeutic candidate for hepatic steatosis in the field of its efficacy and safety.

Overfeeding or consuming an HFD is usually involved in increasing chronic and low-grade inflammation, which is related to obesity, NAFLD, and diabetes in human and rodent animals. Circulating pro-inflammatory cytokines, such as IL-6 and TNF-α, can be promoted by TG deposition in adipose tissue; in turn, the amplifying inflammatory signal contributes to further adiposity and insulin resistance [27]. Many studies have been reported that OLE or OLE-enriched olive leaf extract acts as an antioxidant molecule and attenuates inflammatory response and oxidative stress by inactivating the NF-κB signaling pathway [15,28,29]. OLE is a natural phenolic compound that abundantly present in daily foods including olives and other plants species like *Jasminum polyanthum* Franch., *Fraxinus angustifolia* Vahl., *Ligustrum ovalifolium* Hassk., and *Leucanthemum vulgare* (Vaill.) Lam. Our previous studies found that the petroleum ether extract of *J. grandiflorum* flowers, which enriched OLE, could ameliorate the inflammation in both 12-O-tetradecanoylphorbol-13-acetate (TPA)-induced mouse skin and HCl/EtOH-induced gastric mucosa in mice by inhibiting the NF-κB signal pathway and its downstream pro-inflammatory effectors IL-6 and TNF-α [9,30]. In line with these findings, the present study found that treatment with OLE-JGF significantly decreased the mRNA expressions of markers of low-grade inflammation including TNF-α, CD68, and MCP-1 in HFD-fed mice. These results indicate that OLE-JGF also exhibited a good anti-inflammatory effect in HFD-fed mice. Consistent with the improvement of inflammation, the administration of OLE-JGF produced a beneficial effect on the reduction of body weight gain and circulating ALT, AST, TG, TC, and LDL-C levels in HFD-fed mice. Furthermore, OLE-JGF treatment also clearly decreased the liver weight and hepatic TG, suggesting OLE-JGF induced a lipid-lowering function. In line with these alterations in vivo, OLE significantly reduced TG level and the number of lipid droplets in OA-induced cells. Taken together, these results suggest that OLE-JGF could significantly reduce hepatic low-grade inflammation and lipid accumulation, thus exhibiting robust metabolic protective effects in hepatic steatosis induced by HFD feeding. These findings were in lined with the previous observations that treatment of OLE could significantly relieve HFD-induced hepatic steatosis and prevent the progression from steatosis to steatohepatitis and fibrosis in the liver of HFD and MCD diet-fed mice [12,13,14]. However, further verification is needed to determine whether OLE is the main contributor to the anti-obesity effect of OLE-JGF.

Current health recommendations rely on the fact that the fundamental cause of obesity and overweight is an energy imbalance between calorie consumption and calorie expenditure. In the present study, daily feeding OLE-JGF reduced the energy uptake in HFD-fed mice to a certain extent, indicating that OLE-JGF could regulate energy homeostasis in mice. This is in agreement with the report of Rocchetti et al., who demonstrated that OLE might modulate gut microbiota and decrease satiety signal, leading to the inhibition of intestinal energy uptake [31]. On the other hand, all mammalians have the capacity to synthesize fatty acids de novo using glucose as the precursor to form TG and store in fat depots [32]. SREBP-1c is primarily responsible for the expression of genes including FAS and ACC, involved in de novo lipogenesis in the liver [32]. In accordance with the previous study [33], we found that the HFD diet promoted the expression of lipogenic genes. Excitingly, the mRNA expressions of hepatic-lipogenesis-related genes (LKB1, SREBP-1c, FAS, and ACC) were markedly inhibited after OLE-JGF supplementation in HFD-fed mice, being similar to the previous reported effects of OLE on lipogenic genes in cholesterol-fed rats [34]. Consistent with the observations in vivo, OLE treatment significantly increased the mRNA expression of LKB1 and decreased the mRNA level of ACC in OA-induced cells. Notably, it is known that SREBP-1c is downregulated by activating AMPK, which in turn inhibits glucose and fatty acid uptake in adipocytes, consequently exerting an anti-lipogenic effect. However, OLE-JGF feeding did not alter the expression of AMPK phosphorylation and total AMPK in the liver of HFD-fed mice, which was inconsistently with attenuated SREBP-1c expression, indicating that the inhibition of hepatic lipogenesis and anti-obesity effects of OLE-JGF in HFD-fed mice may not be required for the involvement of AMPK signaling pathway. This finding differs from previous reports in which OLE alleviates metabolic phenotype by activating AMPK signaling or AMPK-dependent autophagy in gestational diabetes mellitus and NAFLD mice [35,36], as well as inducing insulin sensitivity in C2C12 muscle cells [37]. This might help to explain that the attenuation of hepatocellular lipid dysmetabolism induced by OLE-JGF may be related to the novel molecular mechanisms, which are needed to be well explored.

LKB1, a major upstream kinase of AMPK, has been suggested as a key regulator of energy metabolism through AMPK and 12 other closely related kinases [38]. Loss of AMPK does not alter the regulation of hepatic gluconeogenesis, but ablation of hepatic LKB1 was associated with hyperglycemia in mice [39]. Similarly, in the present study, OLE-JGF supplementation not only significantly reduced the levels of fasting blood glucose but also upregulated LKB1 without activating AMPK in both liver from HFD-fed mice and OA-treated cells. Notably, treatment with OA induced a significant decrease in the LKB1 mRNA expression and increase TG accumulation in cells, which were effectively reversed by the activation of LKB1 using both OLE and LKB1 activator metformin. These results indicated that activation of LKB1 but not AMPK may be conducive to the attenuation of the hepatocellular lipid dysmetabolism induced by OLE-JGF. This is also supported by previous report that activation of LKB1 has been shown to improve obesity-associated metabolic disorders by promoting energy expenditure [40]. In line with the effect on LKB1, hepatic PGC-1ɑ was markedly increased in OLE-JGF-treated mice. Notably, PGC-1ɑ functions as an essential regulator for mitochondrial biogenesis and energy metabolism, which plays an important role in the prevention of dyslipidemia and NAFLD [41,42]. The activation of PGC1α has been shown to regulate several transcription cofactors including Nrf2, Tfam, and UCP2. Nrf2 and Tfam regulate the transcription of key mitochondrial enzymes and mtDNA synthesis to enhance mitochondrial capacity, which provides benefits for various metabolic disorders [43,44]. Several experimental and clinical studies revealed that metabolic disorder induced by HFD is accountable for the mitochondrial dysfunctions and decreased number of mitochondria leading to lipid accumulation in adipocytes [45]. Consistently, we found that the relative mRNA expressions of mitochondrial-biogenesis-related genes PGC-1α, Tfam, and Nrf2, as well as mtDNA, were dramatically downregulated in the liver from HFD-induced obese mice. Interestingly, OLE-JGF treatment led to the increased transcriptions of these nuclear genes related to mitochondrial biogenesis and function, as illustrated by the significant upregulation of the hepatic mRNA expressions of these markers of mitochondrial-biogenesis-related genes and the thermogenic gene UCP2 in OLE-JGF-treated mice. Furthermore, OLE also activated the mRNA expressions of PGC-1α and Tfam in OA-induced cells with the inhibition of LKB1. Importantly, we also identified PGC-1α as a critical downstream gene of LKB1 from the ChIP results that LKB1 was directly enriched in the promoter of PGC-1α gene in the liver in HFD-fed mice and OA-treated cells. These results reveal that the LKB1-PGC-1α-pathway-mediated mitochondria biogenesis may be indispensable for the amelioration of hepatocellular lipid dysmetabolism induced by the OLE-JGF largely independent bypass AMPK axis. However, the detailed mechanism underlying the LKB1-PGC-1α axis activated by OLE-JGF has not been explored thoroughly, which is the evident limit of the present study. Taken together, these findings indicate the key role of the OLE-JGF-induced activation of the LKB1-PGC-1α axis and its resultant effects against HFD-induced hepatic steatosis in mice. The LKB1-PGC-1α axis mediates hepatic lipogenesis, and mitochondrial biogenesis may be expected to serve as a promising therapeutic target in hepatic steatosis.

## 5. Conclusions

**Figure 7:**
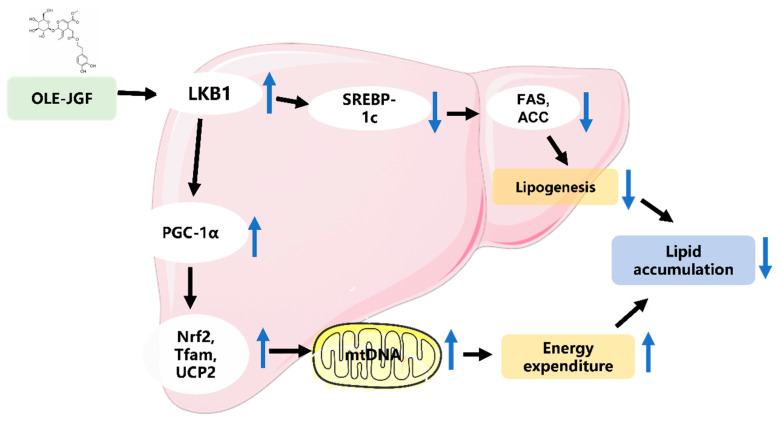
Proposed mechanism by which OLE-JGF protects against hepatocellular lipid dysmetabolism. OLE-JGF may downregulate the expression of SREBP-1c by the activation of LKB1, resulting in the downregulation of FAS and ACC, thereby inhibiting lipogenesis. On the other hand, LKB1 may directly activate PGC-1α to enhance the expressions of Nrf2, Tfam, and UCP2, which promote energy expenditure.

In summary, dietary OLE-JGF could prevent the development of hepatocellular lipid dysmetabolism triggered by HFD in mice or OA in liver cells. OLE-JGF improves metabolic dysfunction involving the inhibition of hepatic lipogenesis and activation of mitochondrial biogenesis, which may be mediated by the LKB1-PGC-1α axis (Figure 7). These findings offer novel insights into the underlying mechanisms by which OLE-JGF affects the LKB1-PGC-1α signaling pathway involved in modulating lipid metabolism, as well as supporting the potential use of OLE-JGF as a supplement for minimizing the risks of various metabolic diseases.

## Data Availability

The original contributions presented in the study are included in the article/Supplementary Materials; further inquiries can be directed to the corresponding authors.

## Supplementary Materials

The following supporting information can be downloaded at: https://www.mdpi.com/article/10.3390/nu16010058/s1, Figure S1: The standard curves for determining triglycerides in tissues and cells. (A) Detection of hepatic triglycerides in mice. (B) Detection of triglycerides in the AML-12 cells. Table S1: Sequences of primers used for the quantitative RT-PCR. Table S2: Sequences of primers used for determining mitochondrial DNA (mtDNA) copy numbers. Table S3: Sequences of primers used in ChIP.

## Abbreviations

ACC, acetyl-CoA carboxylase; ALT, alanine aminotransferase; AML-12, alpha mouse liver 12; AST, aspartate transaminase; CD68, cluster of differentiation 68; CHO, cholesterol; EDTA, ethylenediaminetetraacetic acid; FAS, fatty acid synthase; GAPDH, glyceraldehyde 3-phosphate dehydrogenase; gWAT, gonadal white adipose tissue; H&E, hematoxylin and eosin; HFD, high-fat diet; HPLC, high performance liquid chromatography; HPLC-DAD, HPLC with diode-array detection; IHC, immunohistochemistry; LDL-C, low-density lipoprotein cholesterol; LKB1, liver kinase B1; MCP-1, monocyte chemoattractant protein-1; MetS, metabolic syndrome; mRNA, messenger RNA; mtDNA, mitochondrial DNA; Nrf2, nuclear factor-erythroid-derived 2-like 2; OLE-JGF, oleuropein-rich extract from *Jasminum grandiflorum* L. flowers; OLE, oleuropein; PCR, polymerase chain reaction; PGC-1α, peroxisome proliferator–activated receptor-γ coactivator-1α; SEM, standard error of mean; SREBP-1c, sterol regulatory element-binding protein; TC, total cholesterol; Tfam, mitochondrial transcription factor A; TG, total triglyceride; TNF-α, tumor necrosis factor-α; UCP2, uncoupling protein 2.
