# Supplementary material for: Oleuropein-Rich Jasminum Grandiflorum Flower Extract Regulates the LKB1-PGC-1α Axis Related to the Attenuation of Hepatocellular Lipid Dysmetabolism
Source: Nutrients. 2023 Dec 24;16(1):58. doi: 10.3390/nu16010058 (PMC10780778; doi:10.3390/nu16010058)
Supplement: Supplementary file 1 [file nutrients-16-00058-s001.zip › nutrients-2723036-supplementary.pdf]

Supplementary Materials

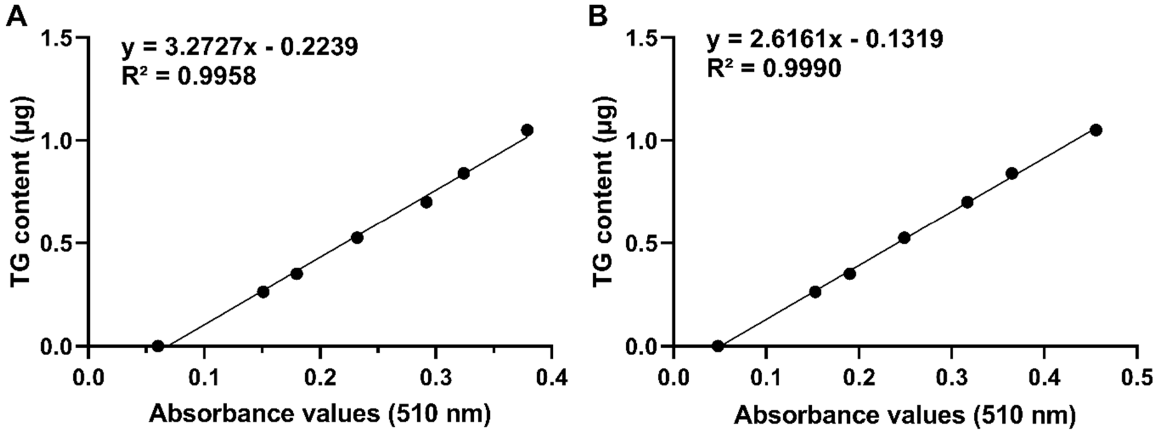

Supplementary Figure S1. The standard curves for determining triglycerides in tissues and cells. (**A**) Detection of hepatic triglycerides in mice. (**B**) Detection of triglycerides in the AML-12 cells.

Supplementary Table S1. Sequences of primers used for the quantitative RT-PCR.

Genes	Sequence 5'-3'	Sequence 3'-5'
UCP2	ATGGTTGGTTTCAAGGCCACA	CGGTATCCAGAGGGAAAGTGAT
FAS	ATTGGCTCCACCAAATCCAAC	CCCATGCTCCAGGGATAACAG
SREBP-1c	CAGCTCAGAGCCGTGGTGA	TGTGTGCACTTCGTAGGGTC
ACC	AGGATTTGCTGTTTCTCAGAGCTT	CAGGATCTACCCAGGCCACAT
LKB1	TGGCTATGAGGATGTTTGGA	GTGGTTGGTCGTGTTGTCTG
PGC-1α	GCCTATGAGCACGAAAGGCT	GCGCTCTTCAATTGCTTCT
Nrf2	ACACAGCATAGCCCATCTCGT	ACCAACCTGGATGAGCGACAC
TFAM	CACCCAGATGCAAAACTTTCAG	CTGCTCTTTATACTTGCTCACAG
CD68	GCTACATGGCGGTGGAGTACAA	ATGATGAGAGGCAGCAAGATGG
TNF-α	TCTCATGCACCACCATCAAGGACT	ACCACTCTCCCTTTGCAGAACTCA
MCP-1	ATGCAGGTCTCTGTCACG	CTAGTTCTCTGTCATACT
β-actin	TGGAATCCTGTGGCATCCATGAAA	TAAAACGCAGCTCAGTAACAGTCC

Supplementary Table S2. Sequences of primers used for determining mitochondrial DNA (mtDNA) copy numbers.

Genes	Sequence 5'-3'	Sequence 3'-5'
18S rRNA	AACTTTCGATGGTAGTCGC	TTCCTTGGATGTGGTAGCC
Cyto b	GGCTACGTCCTTCCATGAGG	TGGGATGGCTGATAGGAGGT

Supplementary Table S3. Sequences of primers used in ChIP.

Genes	Sequence 5'-3'	Sequence 3'-5'
PGC-1α Promoter	TCGGGGGTGTTGCCTTCAAACAC	AGCTTGGAACTGAAGCAAGCGCTCC
